# An innovative methodology in analyzing certain pendulum oscillators

**DOI:** 10.1038/s41598-025-99645-x

**Published:** 2025-05-07

**Authors:** Galal M. Moatimid, T. S. Amer, Abdallah A. Galal

**Affiliations:** 1 Department of Mathematics, Faculty of Education, Ain Shams University, Cairo, Egypt; 2 Department of Mathematics, Faculty of Science, Tanta University, Tanta, 31527 Egypt; 3 Department of Engineering Physics and Mathematics, Faculty of Engineering, Tanta University, Tanta, 31734 Egypt

**Keywords:** Simple pendulum, Nonlinear oscillations, Non-perturbative approach, He’s frequency formula, Stability diagrams, Phase portraits, Mechanical engineering, Applied mathematics

## Abstract

Pendulum oscillators study harmonic motion, energy conservation, and nonlinear dynamics, providing insights into mechanical vibrations, wave phenomena, weather patterns, and quantum mechanics, with real-world applications in engineering, seismology, and clock mechanisms. The present study addresses three distinct issues related to SPs; a charged magnetic spherical simple pendulum (SP), and a SP composed of heavy cylinders that roll freely in a horizontal plane, and a nonlinear model depicting the motion of a damped SP in a fluid flow. The SPs are analyzed via an innovative technology known as the non-perturbative approach (NPA), which is based on He’s frequency formula (HFF). This advanced approach linearizes a nonlinear ordinary differential equation (ODE), enabling more straightforward analysis and solution. As-well known, implementing the NPA has several advantages, chief among them the removal of the constraints associated with managing Taylor expansions. Consequently, there have been no augmentations to the current restorative forces. Secondly, the novel method enables us to assess the stability criteria of the system away from the traditional perturbation techniques. The numerical comparison of nonlinear ODEs into linear ones using Mathematica Software (MS) is conducted to validate this innovative method. An analysis of the two responses demonstrates a strong concordance, underscoring the necessity of precision of the methodology. Furthermore, to demonstrate the influence of the components on motion behavior, the time history of the calculated solution and the corresponding phase plane plots are accumulated. The use of multiple phase portraits aims to explore stability and instability near equilibrium points by examining the interaction between expanded and cyclotron frequencies, modulated by the magnetic field, for varying azimuthal angular velocities.

## Introduction

The study of SP is driven by the increasing request to improve dynamical system performance, with a focus on applications like vibration control and energy harvesting in diverse industries. An SP is described as a point mass attached to an inextensible thread and the string possesses minimal mass. The entire system is considered in the presence of gravitational force, which facilitates the SP’s oscillation in the vertical plane. The mass may also fluctuate due to variations in gravitational force at different locations^1–3^. In engineering applications, SPs are particularly beneficial for fresh explanations. They represent a physical process, analogous to condensed instructional materials for spaceflight and manufacturing^4^. This work employed nonlinear control theory to regulate the inverted pendulum^5^. Taylor expansion was utilized to help in the study due to the incorporation of the restoring forces. The physics of the rotating technique of the simple gravity pendulum is examined, together with the application of the direct method in physics to describe these equations, as well as the discussion of numerical solutions (NS)^6^. The SP serves as a fundamental classical example of simple harmonic motion. The amplitude of an SP oscillating in air progressively reduces as its mechanical energy is eventually dissipated due to air resistance^7^. The developed digital SP prototype demonstrates that the period and frequency are independent of the object’s mass and are depending upon the string’s length. A report allowed pupils to deepen their understanding^8^. The SP serves a vital function in the dynamics as a conventional physical model. Nevertheless, the majority of research concentrates on optimal SP structure, which diverges from actual conditions. The SP was analyzed in practical contexts^9^. This work demonstrated the application of a tracker in relation to a basic SP problem^10^.

Nonlinear dynamics examines systems that exhibit temporal fluctuations. Their applications duration physics, celestial mechanics, and practical uses like oscillating structures, compressors, satellites, and transportation systems. Prominent among these issues are those pertaining to celestial mechanics, particularly the examination of the movements of bodies within the solar system^11^. A method of calculating the angular frequency of un-damped nonlinear oscillations was proposed^12^. The newly formulated equation was carefully applied to the cubic-quintic Duffing oscillator (DO), the SP, the capillary oscillator, and various other oscillators with cubic, harmonic, tangent, and hyperbolic tangent restoring forces^13^. An oscillation criterion of generalization of linear and semi-linear Euler difference equations was established using a modified adapted Riccati transformation^14^. Reproduction of computational fluid dynamics was performed to evaluate temporal frequency shifts, comparing two distinct materials with consistent outcomes^15^. The results indicated the possibility of creating correction factors of oscillations with greater starting deformations. The nonlinear analysis of the synchronous reference frame phase-locked loop under unbalanced grid voltage was conducted^16^. The method for calculating the angular frequency of un-damped nonlinear oscillations was streamlined^12^. The notions of complete oscillation, rotation, and wandering, with complete non-oscillation, non-rotation, and non-wandering, were presented for a system of ODEs^17^. A direct relationship was identified between these features and the corresponding characteristics of the system. The forced nonlinear oscillations of a gas bubble in a liquid were examined when the frequency of external pressure variations matched the bubble’s natural frequency^18^. The dynamic response and instability of a system governed by the nonlinear forced Mathieu equation were examined^19^. The multiple time scales method was employed to the third-order approximation, yielding an approximate solution. Meanwhile, modulation equations were established, and the resonance case was analyzed with respect to solvability constraints.

Both original and modified iterations of the HFF were highlighted for their accuracy^20^. A concise summary of amplitude-frequency equations of nonlinear oscillators was presented^21^. A modified version was suggested to improve the accuracy of HFF; two examples were presented, illustrating that the resulting solutions displayed remarkable precision and are applicable over the whole solution domain^22^. The HFF actively participates in the examination of nonlinear conservative oscillators. The formulation can be extended to incorporate damping scenarios, producing findings equivalent to those obtained via the homotopy perturbation method (HPM)^23^. Moatimid et al.^24–27^ utilized the HPM to analyze various issues in mechanics as well as Dynamical Systems. The frequency-amplitude relationship of a nonlinear oscillator in HFF can be established using the residuals from two experimental solutions^28^. This approach produces a highly accurate result; however it has potential for further enhancement. The HFF, originating from an old Chinese algorithm, is an efficient method for approximating solutions of nonlinear oscillators. A more direct formulation was suggested based on HFF^29^. The HFF of nonlinear oscillators was introduced and validated, along with a proposed modification^30^. The DO, noted for its considerable nonlinearity, functions as a scientific model to illustrate the solution approach and accuracy. Moreover, the NPA serves as a unique and unequivocal alternative to traditional perturbation techniques in the domain of nonlinear oscillating systems. This method is a robust mathematical instrument capable of addressing a diverse array of parameter conditions, particularly those marked by considerable nonlinearity. Significant advancements of the NPA as a crucial method for producing analytical approximations in nonlinear oscillator studies were documented^31–43^. The aim of the NPA is to reduce the complexity of the nonlinear model, making it more controllable and allowing for clear and precise solution specification. Implementing the NPA yields a more accurate assessment of the original system’s behavior within the designated range. Consequently, rather than depending on the iterative alteration of perturbation methods, the NPA introduced an innovative viewpoint. The NPA allows for a deeper exploration of a broader range of system behaviors, undisturbed by small disruptions.

The experimental and practical applications of SPs studies span various scientific and engineering fields. In laboratories, reliable measurements of a pendulum’s period facilitate precise calculations of gravitational acceleration, a fundamental constant in physics. By manipulating factors like as length, mass, and damping forces, researchers acquire insights into harmonic motion, energy transfer, and resonance, which are critical in mechanical and structural engineering. In addition to theoretical studies, pendulums have substantial practical applications: they function as the fundamental mechanism in pendulum clocks, establishing a historical basis for timekeeping precision. In seismology, big pendulums, like seismographs, identify and document earthquake vibrations, facilitating early warning systems. Engineers and architects utilize pendulum principles in the construction of earthquake-resistant structures, employing tuned mass dampers to mitigate oscillations induced by seismic activity or wind forces. Gyroscopic pendulums play a crucial role in stabilizing satellites, aircraft, and submarines within aerospace and navigation. In quantum physics, the examination of microscopic pendulum-like oscillators aids in comprehending wave-particle duality and fundamental interactions. The extensive relevance of pendulum research illustrates their lasting significance in scientific inquiry and technological progress. The subsequent details should be emphasized concerning the new technique utilized or the remarkable results achieved:

1. The employed method generates an analogous linear ODE that corresponds to the existing nonlinear one.
2. This procedure results in a precise correspondence between the two ODEs.
3. The traditional perturbation techniques use Taylor expansion to simplify problems involving restoring forces. The methodology presented here addresses this gap.
4. Unlike other traditional methods, the NPA allows for the assessment of the problem’s stability analysis.
5. The original method presents itself as a straightforward, valuable, and engaging tool, applicable to the analysis of multiple types of nonlinear oscillators.

To facilitate presenting, the current material has been split into five sections. The NPA is used in **§ 2** to study three nonlinear ODEs from three distinct SPs. The SP that connected with a rolling wheel is presented in **§ 3**. The SP that moves in a fluid is presented in **§ 4**. The previous section includes, also, the analysis of time history and phase plane of the given SPs. The main key outcomes are summarized in **§ 5**.

## Formulation of the first problem

The current study employs a crucial method known as the NPA to derive analytical approximation solutions for highly nonlinear ODEs. This article examines three distinct SP oscillators. The findings are compared with NS to demonstrate the usefulness and accuracy of the existing method. The current method yields findings that are more precise than comparable estimates for the aforementioned concerns. The NPA possesses significant potential and can be employed to address other highly nonlinear issues, as proven in the conclusion. The NPA often addresses several real occurrences. To address these real-world challenges, established analytical methodologies published in the literature are employed. However, optimal results are achieved more rapidly using our current technique. Nonetheless, because to the NPA’s comprehensive representation of the processes necessary for grasping the analytical solutions, the computations performed with MS are more straightforward than those associated with alternative analytical approaches. Alternative computational methods were time-consuming or challenging to employ.

**Fig. 1:**
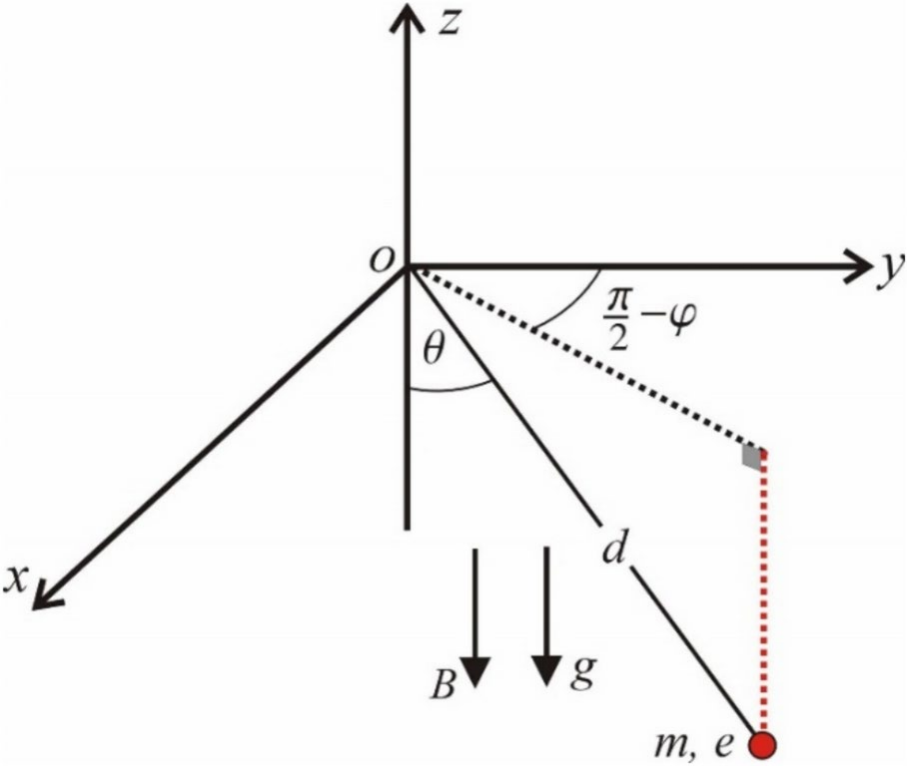
A magnetic charged spherical SP.

The investigation of the magnetic spherical SP has practical implications in numerous scientific and technological domains. It can be utilized to simulate and analyze dynamic systems in geophysics, such as the interactions of the Earth’s magnetic field with hanging objects. It also has ramifications in engineering, especially in the design of magnetic sensors and dampening systems for mechanical apparatus. Moreover, comprehending the pendulum’s dynamics aids in educational and research contexts to demonstrate intricate scientific concepts such as magneto-mechanical coupling and chaotic motion, pertinent to disciplines from robotics to space exploration. Under certain conditions, the equation of motion (EOM) for a magnetic spherical pendulum, shown in Fig. 1, is derived as a differential equation. The current work seeks to develop an analytical, bounded procedure for solving this equation.

Following the derivation of our previous work^1^, the governing EOM:

1 $$\ddot{\theta } + \alpha \sin \theta - \;\beta \sin \theta \cos \theta = 0,$$

where $$\alpha = g/d\;{\text{and}}\;\beta = \kappa^{2} - 2\kappa \;\omega_{c} ;\;\kappa = \dot{\varphi } = {\text{const}}.\;$$

The parameters $\alpha$ and $\beta$ in the governing equation of motion for a charged spherical pendulum, are essential. They represent the physical qualities and interactions to dictate the system’s behavior. Generally, they denote a dimensionless quantity associated with the charge-to-mass ratio or the intensity of the electromagnetic interaction in comparison to gravitational forces, reflecting the influence of the pendulum’s charge on its dynamics within an external field. Conversely, $\beta$ often denotes the ratio of gravitational forces to centripetal forces or other system-specific scaling variables, affecting the trajectory’s shape and motion stability. Collectively, these factors ascertain the relative significance of electrical, gravitational, and inertial forces, influencing the system’s dynamics and equilibrium states in a charged environment. By using the NPA enables us to convert the nonlinear ODE that is given in Eq. (1) into another linear ODE.

To establish a gussing (trial) solution as

2 $$\nu = \nu_{0} \cos Qt,$$

where $Q$ is the total frequency of the system.

with the ICs: $\nu (0) = \nu_{0}$ and $\dot{\nu }(0) = 0$.

Based on the reasoning presented by Moatimid et al.^31–38^, Eq. (1) can be expressed as:

3 $$\ddot{\theta } + F(\theta ) = 0,$$

where

$$F(\theta ) = \alpha \;\sin \theta - \beta \sin \theta \cos \theta .$$

The equivalent frequency may be determined as^31–38^:

4 $$Q^{2} = \int_{0}^{2\pi /Q} {\nu \;F(\nu )dt/\int_{0}^{2\pi /Q} {\nu^{2} \;dt.} }$$

By making use of the MS, Eq. (4) produces

5 $$Q^{2} = \frac{1}{{\nu_{0} }}\left( {2\alpha \;J_{1} (\nu_{0} ) - \beta J_{1} (2\nu_{0} )} \right),$$

where the functions $J_{1} (\nu_{0} )$ and $J_{1} (2\nu_{0} )$ are the Bessel functions of the first kind.

Therefore, the comparative linear differential equation is given as:

6 $$\ddot{\nu } + Q^{2} \;\nu = 0.$$

Therefore, the stability standard requires $Q^{2} > 0.$

**Fig. 2:**
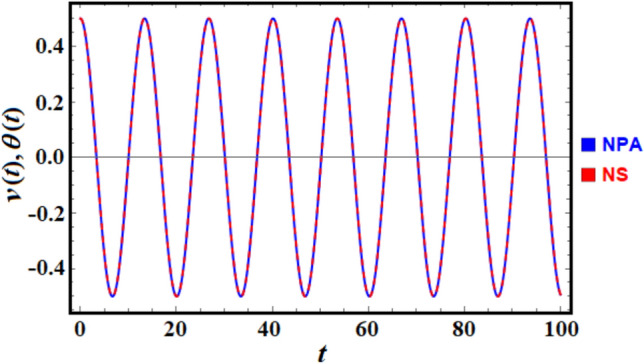
Provides a comparison between the NS and NPA.

To enhance convenience, the *NDSolve* command of the MS facilitates a numerical comparison between the solutions of the nonlinear ODE presented in Eq. (1) and the linear ODE outlined in Eq. (6) as shown in Fig. 2. For this purpose, consider the data:

$$\alpha = 0.5\;,\;\;\;\;\;\beta = 0.3\;\;{\text{and}}\;\;\nu_{0} = 0.5.\;$$

Notably, the MS produces an absolute error of 0.00609.

Table 1 compares the results obtained by NPA with numerical results using the Rung-Kutta fourth order (RK-4). This comparison shows high constancy between them, in which the deviation between them is calculated in this table.

**Table 1:** Comparison between the numerical results and the NPA ones for the first problem.

$t$	Numerical results	NPA results	Absolute error
$0$	$0.5$	$0.5$	$0$
$10$	$- 0.00854797$	$- 0.00828779$	$0.000260183$
$20$	$- 0.49969$	$- 0.499725$	$0.0000354721$
$30$	$0.0256345$	$0.0248543$	$0.000780239$
$40$	$0.49876$	$0.498901$	$0.0001415$
$50$	$- 0.0426928$	$- 0.0413934$	$0.00129943$
$60$	$- 0.497211$	$- 0.497529$	$0.000317816$
$70$	$0.0597043$	$0.0578871$	$0.00181727$
$80$	$0.495047$	$0.49561$	$0.000563351$
$90$	$- 0.0766504$	$- 0.0743171$	$0.00233328$
$100$	$- 0.492269$	$- 0.493146$	$0.000877516$

**Fig. 3:**
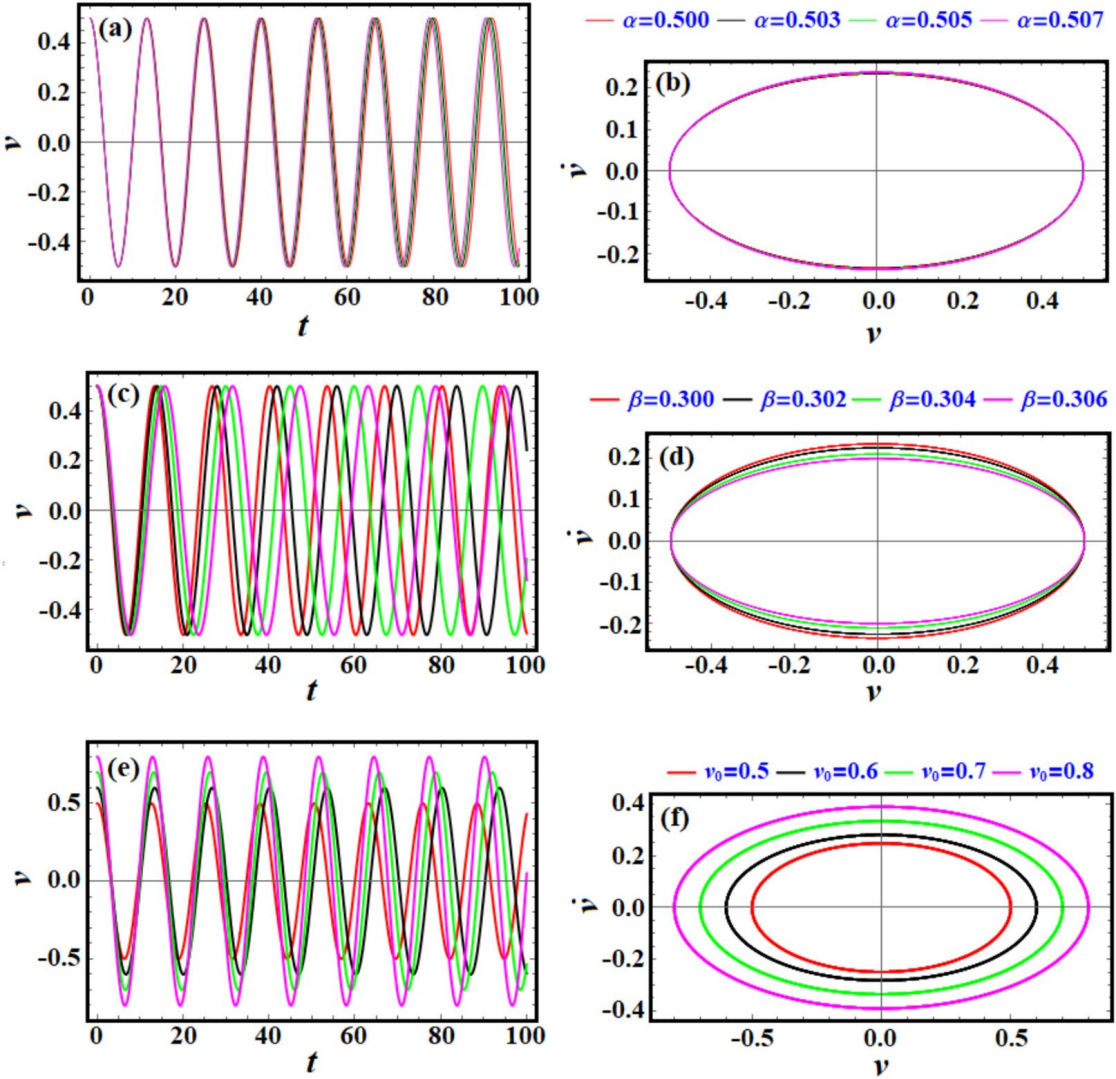
(**a**), (**c**), and (**e**) present the time variation of $v$ according to Eq. (6), while (**b**), (**d**), and (**f**) are the corresponding curves in the pane $(\nu - \dot{\nu }).$

Figure 3 shows both the time evolution of the periodic solution of Eq. (6), as in portions (a), (c), and (d), and the related phase-plane diagrams, presumably describing the relation between the velocity and displacement and in the other portions of this figure. Figure 3a shows how the parameter $\alpha$ influences the system’s behavior over time. The drawn periodic curves for the mentioned values of $\alpha$ indicate a growing variation in the amplitude and frequency of oscillations. As $\alpha$ increases, the oscillations’ amplitude or frequency slightly changes. Portion (c) describes the evaluation of the solution based on the given values of parameter $\beta$. The effect on the oscillatory behavior of $\nu$ is shown over time. Changes in $\beta$ seem to affect both amplitude and frequency. Higher $\nu_{0}$ leads to larger amplitudes, indicating sensitivity of the solution to the values of $\nu_{0}$, as seen in Fig. 3e. The presented curves in the other portions (b), (d), and (f) show the phase plane plots that plot $\nu$ against its first derivative $\dot{\nu }$ for different values of the mentioned parameters. The trajectories form closed curves, indicating periodic motion. As these parameters change, these curves expand or shift, implying changes in energy or stability characteristics. The closed nature of the trajectories implies sustained periodic motion, consistent with nonlinear oscillatory systems.

**Fig. 4:**
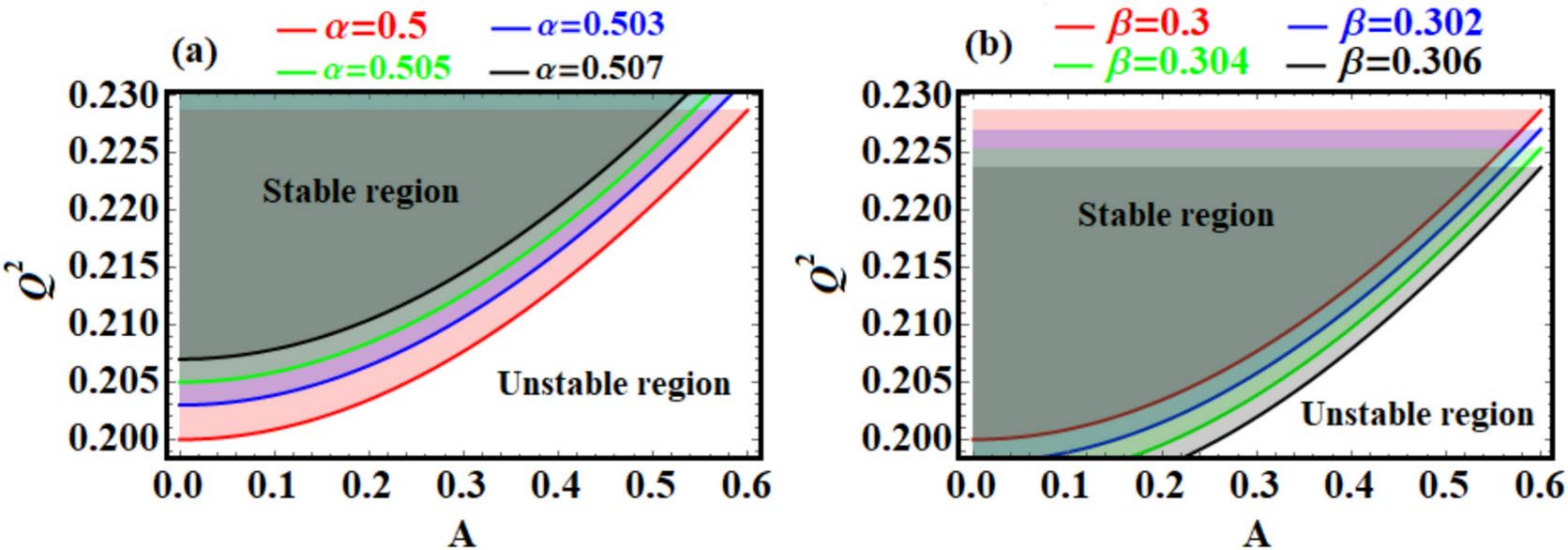
Illustrates the stability curves of Eq. (5) for various values of: (**a**) $\alpha$, and (**b**) $\beta$ .

Figure 4 appears to depict stability regions and curves for the considered system, as in Eq. (5). The panels represent the relationship between system parameters $$(Q^{2} ,\;\nu_{0} ,\;\alpha ,\;\;{\text{and}}\;\beta )$$ and their influence on the stability behavior. The plotted curves in Fig. (4a) show the relationship between $$Q^{2}$$ (possibly a frequency) and $$\nu_{0}$$(likely a measure of amplitude or excitation level) for different values of $$\alpha$$$$(\alpha = 0.5,\;\;0,503,\;\;0,505,\;\;0.507)$$. The shaded regions represent stable regions of the system, while outside they could indicate instability. As $$\alpha$$ increases, see Fig. 4a, the stability boundary shifts upward (toward higher $$Q^{2}$$) for a given $$\nu_{0}$$. This suggests that higher $$\alpha$$ values make the system more resistant to instability, possibly due to stronger restoring forces or reduced sensitivity to excitation. The curves are nonlinear and exhibit a gradual increase with $$\nu_{0}$$, highlighting the interplay between amplitude and stability. The shaded region is narrower for lower $$\alpha$$, indicating reduced stability margins compared to higher $$\alpha$$.

The presented stability curves for varying $\beta$, as in Fig. 4b, show the relationship between $Q^{2}$ and $\nu_{0}$ for different values of $\beta$ $(\beta = 0.3,\;0.302,\;0.304,\;0.306).$ Similar to panel (a), the shaded region indicates stability, while the area outside could signify instability. As $\beta$ increases, the stability boundary shifts downward slightly, suggesting increased stability for higher $\beta$. However, the effect of $\beta$ is subtler compared to $\alpha$ in panel (a), as the curves are closely spaced. The nonlinear trend of the stability boundary is consistent, indicating similar dynamics to those in panel (a). The shaded region’s width indicates stability is maintained over a broad range of $\nu_{0}$ and $\beta$ but decreases as $\beta$ reduces. Stability and instability are essential ideas with significant implications across several disciplines, including physics, engineering, economics, and social systems. Stability denotes a system’s capacity to sustain or revert to equilibrium when faced with perturbations, hence assuring predictability, order, and resilience. This is crucial in structures, ecosystems, financial markets, and governance, as it facilitates sustainable operation and development. Instability denotes a system’s susceptibility to disturbances, frequently resulting in swift transformation, disorder, or disintegration. Although instability may provide problems, it can also catalyze innovation and change when systems adapt or evolve in reaction to external influences. Collectively, these notions underscore the intricate equilibrium necessary to sustain harmony while promoting advancement in dynamic settings.

## Outline of the second problem

**Fig. 5:**
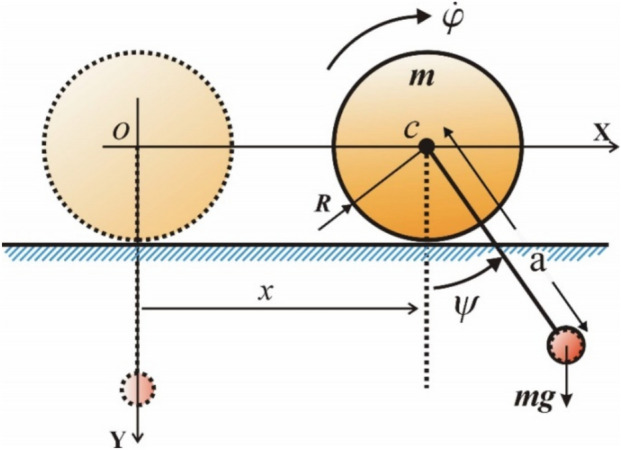
Depicts a cylinder of mass rolling without slipping on a horizontal table, with an attached pendulum.

Looking at the combined system in Fig. 5, which is made up of a cylinder with mass $m$ that rolls smoothly on a flat surface and has a pendulum attached to its axis that moves back and forth freely because of gravity. Examining the dynamics of a cylinder rolling without slipping on a horizontal surface, with its axis connected to a pendulum oscillating under gravitational influence, has practical implications for comprehending energy transfer and rotational motion in mechanical systems. This configuration functions as a framework for examining integrated translational and rotational dynamics, essential in designing mechanisms such as gyroscopes, robotic arms, and stabilization systems. Furthermore, it offers insights into practical applications, like the motion of rolling drums in manufacturing, pendulum-driven timekeeping mechanisms, and the stabilization of wheeled vehicles on irregular terrains, illustrating the interaction between oscillatory motion and rotational limitations. The pendulum bob, with mass $m$, matches the mass of the cylinder, and the length of the pendulum rod is $a$, treated as massless. The generalized coordinates are the horizontal displacement $x$ of the cylinder and the angle $\psi$ of the pendulum rod from the vertical. The cylinder has a radius $R$ and a homogeneous mass distribution.

Using the following ICs: at $\dot{x}(0) = 0,\;\dot{\psi }(0) = 0\;$ and $\psi (0) = \psi_{0} \ne 0.$

The two Lagrange’s equations of this system will be:

$$A=\pi {r}^{2}$$

7 $$\begin{gathered} \frac{d}{dt}\left( {\frac{\partial L}{{\partial \dot{x}}}} \right) - \frac{\partial L}{{\partial x}} = \frac{5m}{2}\ddot{x} + a\ddot{\psi }\;m\;\cos \psi - \;a\;\dot{\psi }^{2} m\;\sin \psi = 0, \hfill \\ \frac{d}{dt}\left( {\frac{\partial L}{{\partial \dot{\psi }}}} \right) - \frac{\partial L}{{\partial \psi }} = m\;a\;\ddot{x}\cos \psi + m\;a^{2} \;\ddot{\psi }\; + m\;g\;a\;\sin \psi = 0. \hfill \\ \end{gathered}$$

From the previous system, it is easy to get the governing EOM as follows:

8 $$\left( {1 - \frac{2}{5}\;\cos^{2} \psi } \right)\ddot{\psi } + \frac{2}{5}\;\dot{\psi }^{2} \cos \psi \;\sin \psi + \frac{g}{a}\;\sin \psi = 0\;.$$

Now, Eq. (8) may be written as:

9 $$\ddot{\psi } + \Phi (\psi ,\dot{\psi },\ddot{\psi }) = 0,$$

where

$$\Phi (\psi ,\dot{\psi },\ddot{\psi }) = \frac{2}{5}\;cos\psi (\dot{\psi }^{2} sin\psi - \ddot{\psi }\;cos\psi ) + \frac{g}{a}sin\psi .$$

The guessing solution may be formulated as:

10 $$\xi = \xi_{0} \cos \Gamma t.$$

where $\Gamma$ is the total frequency of the system.

Considering the ICs:$\xi (0) = \xi_{0} ,\;{\text{and}}\;\;\dot{\xi }(0) = 0.$

The equivalent frequency is determined as^31–38^:

11 $$\Gamma^{2} = \int_{0}^{2\pi /\Gamma } {\xi \;\Phi (\xi ,\dot{\xi },\ddot{\xi })dt/\int_{0}^{2\pi /\Gamma } {\xi^{2} \;dt.} }$$

By making use of the MS, the equivalent frequency may be simplified as:

12 $$\Gamma^{2} = \frac{{10\;g\;J_{1} (\xi_{0} )}}{{a\;\xi_{0} \;(4 - J_{0} (2\xi_{0} )\;)}}$$

where the functions $J_{1} (\xi_{0} )$ and $J_{0} (2\xi_{0} )$ are the Bessel functions of the first kind.

The stability customary needs: $\Gamma^{2} > 0.$

13 $$\ddot{r} + \Gamma^{2} r = 0.$$

To enhance convenience, the NDSolve command of the MS facilitates a numerical comparison between the solutions of the nonlinear ODE and the linear ODE as presented in Eq. (13). Consider the data:

$$a = 5,\;g = 9.8,\;{\text{and}}\;\xi_{0} = 0.4$$.

**Fig. 6:**
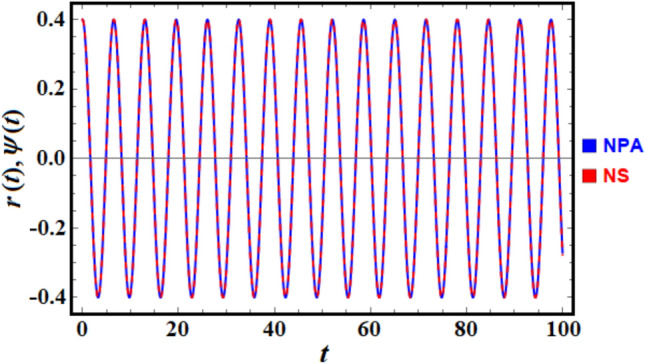
Presents a comparison between the NS and NPA.

Therefore, Fig. 6 is drawn. It is important to assert that the MS produces the absolute error of: 0.00707.

The results obtained by NPA of the second problem are compared with numerical results using the RK-4 method in Table 2. This comparison reveals excellent agreement, with the deviations calculated and displayed in the table.

**Table 2:** Contrasting the numerical results with the NPA results for the second problem.

$t$	Numerical results	NPA results	Absolute error
$0$	$0.5$	$0.5$	$0$
$10$	$- 0.00854797$	$- 0.00828779$	$0.000260183$
$20$	$- 0.49969$	$- 0.499725$	$0.0000354721$
$30$	$0.0256345$	$0.0248543$	$0.000780239$
$40$	$0.49876$	$0.498901$	$0.0001415$
$50$	$- 0.0426928$	$- 0.0413934$	$0.00129943$
$60$	$- 0.497211$	$- 0.497529$	$0.000317816$
$70$	$0.0597043$	$0.0578871$	$0.00181727$
$80$	$0.495047$	$0.49561$	$0.000563531$
$90$	$- 0.0766504$	$- 0.0743171$	$0.00233328$
$100$	$- 0.492269$	$- 0.493146$	$0.000877516$

The intersection of two plane curves, one depicting a linear ODE and the other a nonlinear ODE, is noteworthy as it signifies a unique situation when the behavior of the solutions to these fundamentally distinct equations overlap under particular conditions. This intersection may indicate common solutions or paths, illustrating cases when the nonlinear system resembles linear behavior, potentially due to linearization at equilibrium points or particular symmetries. Such coincidences are essential for comprehending stability, bifurcations, or transitions between linear and nonlinear dynamics, providing insights into the foundational structure of complex systems and facilitating their research in specific areas.

**Fig. 7:**
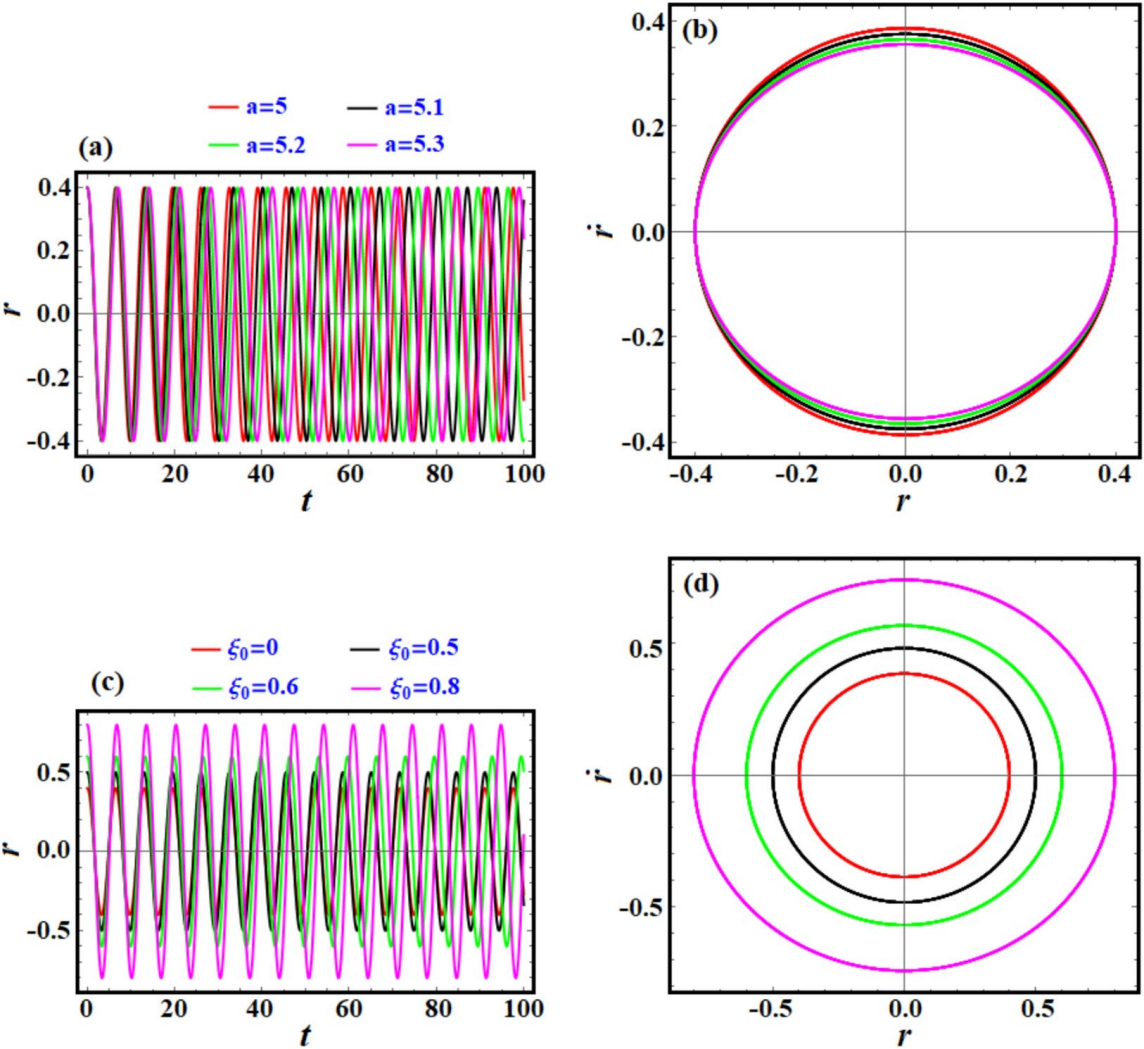
(**a**), (**c**), and (**e**) demonstrate the behavior of the solution $r$ via $t$ as in Eq. (13), while (**b**), (**d**), and (**f**) are the related phase plane curves.

The presented curves in parts of Fig. 7 represent the time evolution and phase-plane dynamics of a system’s radial displacement $r$ under different parameters. In Fig. 7a, the drawn curves show how the parameter $a$ ($a=5, 5.1, 5.2, 5.3$) influences the system’s radial displacement $r$ over time $t$. It is easy to observe that oscillations are periodic for all values of (a), indicating stable periodic motion.

As (a) increases, the amplitude of oscillations increases slightly, and the frequency remains relatively unchanged, suggesting that ${\text{a}}$ affects energy rather than temporal dynamics. Over time, no divergence is observed, indicating that the system does not enter an unstable regime for these values of (a).

Figure 7b shows the phase plots of $r$ against its time derivative $\dot{r}$ for different (a), showing the system’s phase trajectory. The trajectories form closed loops, confirming periodic motion. As ${\text{a}}$ increases, the phase trajectory’s radius expands, consistent with the increasing oscillation amplitude seen in part (a). The shape of the loops remains circular, suggesting linear or weakly nonlinear dynamics.

The time evolution of $r$ for varying $\xi_{0}$ is shown in Fig. 7c describes how varying the parameter $\xi_{0}$ $(\xi_{0} = 0.5,\;0.6,\;0.8)$ affects the system’s radial displacement over time. For increasing $\xi_{0}$, the amplitude of oscillations grows significantly, while the frequency remains stable. Larger $\xi_{0}$ leads more energetic oscillations. Periodicity is maintained across all values of $\xi_{0}$, indicating the absence of instability.

The phase plane ($r$ vs.$\dot{r}$) for varying $\xi_{0}$ is explored in Fig. 7d. This panel shows phase trajectories for $r$ and $\dot{r}$ at different $\xi_{0}$ values. It is noted that the trajectories form concentric loops, with larger $\xi_{0}$ producing larger loops. The symmetry and circular nature of the trajectories are preserved, indicating periodic and stable motion. The increasing size of the loops reflects the growing oscillation amplitude seen in part (c).

**Fig. 8:**
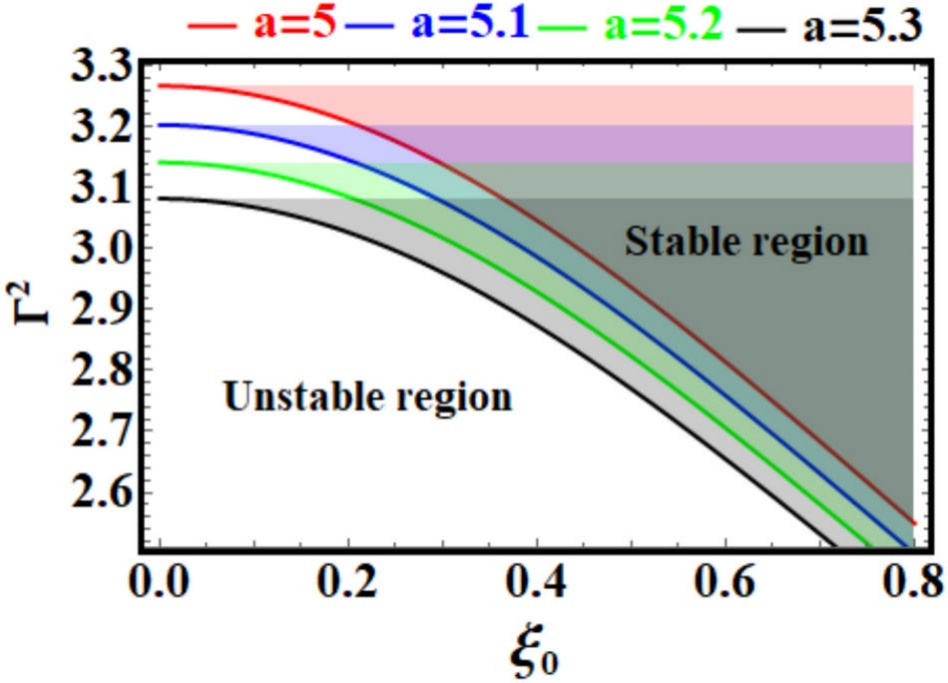
Explores the stability curves for various values of ${\text{a}}$ in light of Eq. (12).

If you look at the curves in Fig. 8, you can see that it shows an analysis of the system’s stability using Eq. (12) and the parameters $\xi_{0}$ and $\Gamma^{2}$. The horizontal and vertical axes are represented by $\xi_{0}$ and $\Gamma^{2}$, respectively. The plot divides the parameter space into stable and unstable regions, with the boundary determined by various curves for specific values of a parameter ${\text{a}}$ (indicated as $a = 5, \, 5.1, \, 5.2, \, 5.3$).

It must be noted that the stable and unstable regions are distinctly separated. The transition occurs along the curves for different values, with $\Gamma^{2}$ decreasing as $\xi_{0}$ increases for each curve. As ${\text{a}}$ increases from $5$ to $5.3$, the stability boundary shifts downward. Higher values lead to a smaller stable region, indicating the system becomes more prone to instability with increasing a. At $\xi_{0} = 0$, $\Gamma^{2}$ starts at its maximum for all curves, suggesting this is the most stable configuration. As ${\xi }_{0}$ increases, $\Gamma^{2}$ decreases monotonically leading to instability at a threshold value. Based on this analysis, one can conclude that adjusting ${\text{a}}$ or controlling $\xi_{0}$ could serve as mechanisms to maintain stability in practical applications like wind turbine blades or pendulum systems. Stability and instability are fundamental concepts having profound consequences across various fields, including physics, engineering, economics, and social systems. Stability refers to a system’s ability to maintain or return to equilibrium when subjected to disturbances, hence ensuring predictability, order, and resilience. This is essential in structures, ecosystems, financial markets, and governance, as it enables sustainable functioning and advancement. Instability refers to a system’s vulnerability to perturbations, sometimes leading to rapid change, chaos, or collapse. While instability may provide challenges, it can also stimulate innovation and transformation when systems adjust or evolve in response to external factors. These concepts highlight the complex balance required to maintain harmony while fostering progress in dynamic environments.

## Clarification of the third problem

**Fig. 9:**
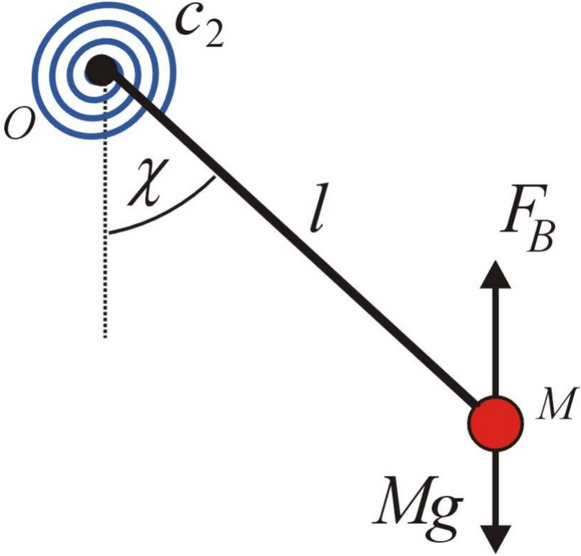
A simple pendulum rotates in a liquid.

Suppose a SP as shown in Fig. 9. At the hinge $O$, there is a spiral spring with a constant of spring $c_{2}$. It resists the rotation of the light rod $OM.$ A small ball $M$ is connected in the end of the pendulum. It moves in a liquid^39^. As-well known, the analysis of a basic pendulum oscillating in a fluid, coupled with a continuous spiral spring, has practical implications across multiple disciplines. The interplay of the SP’s motion, the liquid’s damping effects, and the spring’s restoring force offers insights into fluid dynamics, oscillatory systems, and energy dissipation. These systems are pertinent for the design of shock absorbers, underwater oscillatory devices, and damping mechanisms in engineering. Furthermore, they facilitate the comprehension of natural phenomena such as the behavior of submerged structures in fluids and enhance progress in materials science and mechanical systems where the integration of elastic and fluidic properties is essential.

The generalized force which corresponding to the angle $\chi$ is written as^39^:

14 $$Q_{\chi } = c_{2} \;\dot{\chi } - F_{D} ;$$

where $F_{D}$ is the drag force and equal to:

15 $$F_{D} = \frac{\rho }{2}\;l^{2} \;\dot{\chi }^{2} \;S\;C_{D} ;$$

where.

$\rho$ represents the density of the liquid, $S$ is the cross section of the ball $M$ and $C_{D}$ refers the drag coefficient.

The buoyancy force $F_{B}$ can be represented as: $F_{B} = \rho \,g\,\tilde{Q};$ $\tilde{Q}$ is the volume of the ball $M.$

It is easy to get the equation of motion of the given ball as follows:

16 $$M\left\{ {l^{2} \ddot{\chi } + l\left( {g\; - \;\frac{{F_{B} }}{M}} \right)\sin \chi } \right\} = - \frac{\rho }{2}\;l^{2} \;\dot{\chi }^{2} \;S\;C_{D} \; - c_{2} \dot{\chi }\;$$

The previous equation has been transformed into the following timeless equation:

17 $$\chi \prime \prime + \frac{1}{{l\;\omega^{2} }}\left( {g\; - \;\frac{{F_{B} }}{M}} \right)\sin \chi = - \frac{\rho }{2\;M}A\;C_{D} \;\chi \prime^{2} - \frac{{c_{2} }}{{\omega \;M\;l^{2} \;}}\chi \prime \,;$$

where $$(\prime \equiv \frac{d}{d\tau },\;\tau = \omega t).$$

The equation of the third SP can be derived as follows:

18 $$\chi \prime \prime + \tilde{\alpha }\sin \chi + \tilde{\beta }\,\chi \prime^{2} + \tilde{\gamma }\,\chi \prime = 0.$$

It is evident that the equivalent damping term is $\tilde{\gamma }\,\chi^{\prime}$. Therefore, Eq. (14) may be written as:

19 $$\chi \prime \prime + \tilde{\gamma }\,\chi \prime + \tilde{\psi }\,(\chi ) = - \tilde{\beta }\,\chi \prime^{2} ,$$

where $$\tilde{\psi }(\chi ) = \tilde{\alpha }\sin \chi$$.

The guessing solution may be formulated as:

20 $$\zeta = \zeta_{0} \cos \Delta \tau .$$

Considering the ICs:$\zeta (0) = \zeta_{0} \;,{\text{and}}\,\zeta \prime (0) = 0.$

The equivalent frequency can be written as ^31–46^, one gets:

21 $$\Delta^{2} = \int_{0}^{2\pi /\Delta } {\zeta \tilde{\psi }\left( \zeta \right)d\tau /\int_{0}^{2\pi /\Delta } {\zeta^{2} d\tau = \frac{{2\;\tilde{\alpha }}}{{\zeta_{0} }}J_{1} \left( {\zeta_{0} } \right)} } .$$

Hence, the equivalent linear equation is expressed as follows:

22 $$w\prime \prime + \gamma \;w\prime + \delta^{2} w = - \tilde{\Lambda }.$$

Based on Moatimid et al. ^31–38^, the standard normal form transforms into the following equation:

23 $$\tilde{F}\prime \prime + \Delta^{2} \tilde{F} = - \tilde{\Lambda },$$

where the equivalent frequency is given by

24 $$\Delta^{2} = \delta^{2} - \tilde{\gamma }^{2} /4,$$

where the functions $J_{1} \left( {\zeta_{0} } \right)\;{\text{and}}\;\;J_{0} \left( {2\zeta_{0} } \right)$ are the Bessel functions of the first kind.

The stability customary needs:$\Delta^{2} > 0.$

As shown by Moatimid et al.^31–38^, the constant $\tilde{\Lambda }$ is given as follows:

25 $$\tilde{\Lambda } = \frac{{\zeta_{0}^{2} }}{4}\left( {\delta^{2} - \tilde{\gamma }^{2} /4} \right)\tilde{\beta }.$$

For more convenience, by using the command *NDSolve* of the MS through, a numerical matching between the solutions of the nonlinear ODE as represented in Eq. (18) and the linear ODE as expressed in Eq. (22). Consider the data:

$$\tilde{\alpha } = 0.5,\;\tilde{\beta } = 0.3,\;\tilde{\gamma } = 0.05,\,and\,\zeta_{0} = 0.1\;.$$

**Fig.10:**
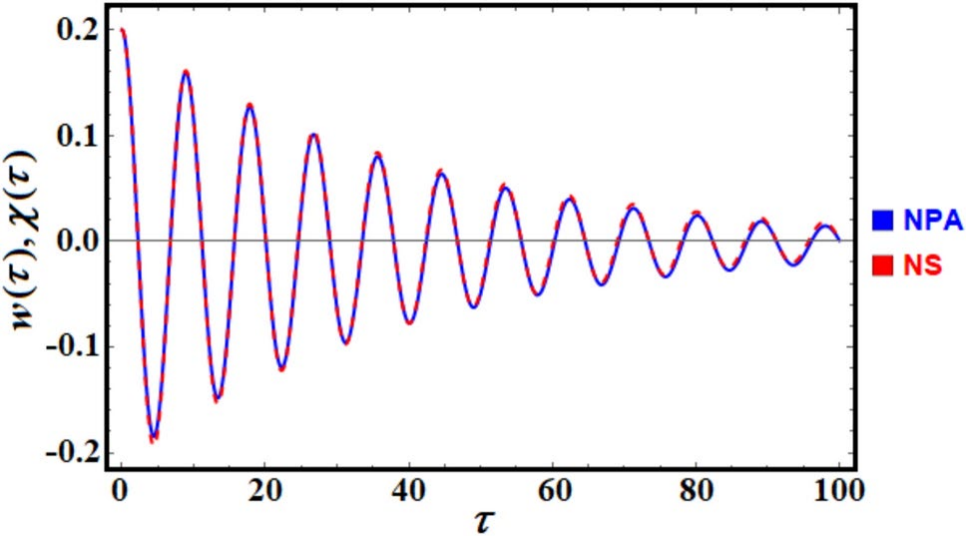
Illustrates a comparison between the NS and NPA.

Therefore, Fig. 10 is drawn. It should be noted that the MS produces the absolute error of the value: 0.0027.

A comparison between NPA results of the third problem and RK-4 numerical results is presented in Table 3, demonstrating strong agreement. The deviations between the two are also calculated and shown in the table.

**Table 3:** Comparison between the numerical results and the NPA ones for the third problem.

$\tau$	Numerical results	NPA results	Absolute error
$0$	$0.2$	$0.2$	$0$
$10$	$0.116861$	$0.114851$	$0.00201087$
$20$	$0.00694197$	$0.00619677$	$0.000745197$
$30$	$- 0.0654494$	$- 0.0641522$	$0.00129719$
$40$	$- 0.0775198$	$- 0.0772372$	$0.000282597$
$50$	$- 0.0462521$	$- 0.0492599$	$0.00300776$
$60$	$- 0.00441971$	$- 0.00991086$	$0.00549115$
$70$	$0.0228952$	$0.0173005$	$0.00559469$
$80$	$0.0278843$	$0.0239866$	$0.00389767$
$90$	$0.0168845$	$0.0149892$	$0.0018953$
$100$	$0.00168317$	$0.000831616$	$0.000851552$

The intersection of two plane curves, one representing a linear ODE and the other a nonlinear one, is significant as it indicates a unique scenario where the behavior of the solutions to these fundamentally different equations coincide under specific conditions. This intersection may signify shared solutions or trajectories, demonstrating instances where the nonlinear system exhibits linear characteristics, possibly as a result of linearization at equilibrium points or specific symmetries. Such coincidences are crucial for understanding stability, bifurcations, or transitions between linear and nonlinear dynamics, offering insights into the fundamental structure of complex systems and aiding study in certain domains.

**Fig. 11:**
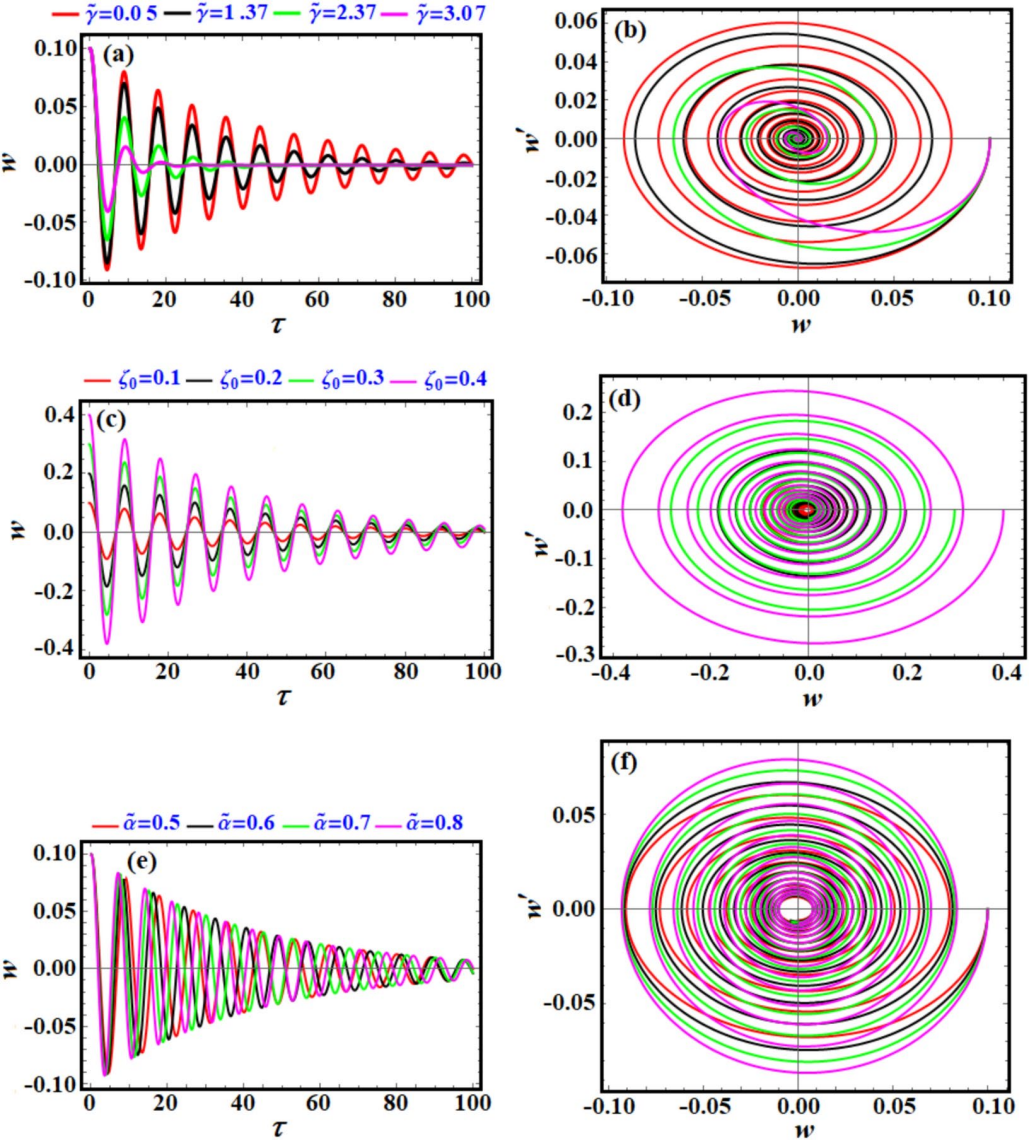
(**a**), (**c**), and (**e**) show the behavior of the solution $w$ according to Eq. (22), while (**b**), (**d**), and (**f**) are the related phase plane curves.

An overview of Fig. 11 shows the dynamic behavior of a system analyzed in two representations: Time-domain plots ($w$ vs. $\tau$) in panels (a), (c), and (e). Phase-space trajectories $(w^{\prime}\,{\text{vs}}{. }\,w)$ in potions (b), (d), and (f). Each row examines the effect of a distinct parameter on the system’s dynamics, in which the top row (a, b) examines the influence of $\tilde{\gamma }$, the middle row (c, d) presents the influence of ${\zeta }_{0}$, while the bottom one (e, f) investigates the impact of $\tilde{\alpha }$.

The time-domain evolution for varying $\tilde{\gamma }$ reveals that the oscillatory behavior is evident for all curves, with different damping intensities; see Fig. 11a. It is observed that as $\tilde{\gamma }$ increases, the amplitude decays more rapidly. In other words, higher $\tilde{\gamma }$ values lead to smoother, less oscillatory behavior over time, indicating stronger damping. The trajectories form spirals, converging to the origin $(w = w^{\prime} = 0)$, as indicated in Fig. 11b. Increased $\tilde{\gamma }$ tightens the spiral, suggesting faster stabilization and stronger dissipation.

Referring to the time-domain evolution for varying $\zeta_{0}$, one can conclude that the graphed oscillations have differing initial amplitudes but exhibit consistent decay rates, as explored in Fig. 11c. Larger $\zeta_{0}$ corresponds to higher initial energy or displacement but does not significantly affect the decay pattern. The plotted curves in Fig. 11d show the phase-plane trajectories for varying $\zeta_{0}$, in which spirals originate further from the origin for higher $\zeta_{0}$, reflecting the system’s initial conditions. All trajectories converge to the origin, implying the system is globally stable regardless of $\zeta_{0}$. Now, we can analyse the graphed curves in Fig. 11e to estimate the time-domain evolution for varying $\tilde{\alpha }$. Increasing $\widetilde{\alpha }$ introduces higher frequency oscillations. Decay of oscillations is similar across all curves, suggesting α primarily affects frequency, not damping. Finally, the corresponding phase-plane trajectories for the presented curves in Fig. 11e are drawn in Fig. 11f. Spirals are tighter and more intricate for larger $\tilde{\alpha }$, consistent with increased oscillation frequencies. Convergence to the origin remains uniform across all curves, indicating stability.

All scenarios converge to the origin, confirming the system is asymptotically stable under the examined parameters. The parameter $\tilde{\gamma }$ is a critical control parameter for reducing oscillations and achieving faster stabilization. The parameter $\tilde{\alpha }$ can be used to tune oscillation frequencies without compromising stability. Moreover, stability is unaffected by $\zeta_{0}$, demonstrating robustness to initial perturbations.

**Fig. 12:**
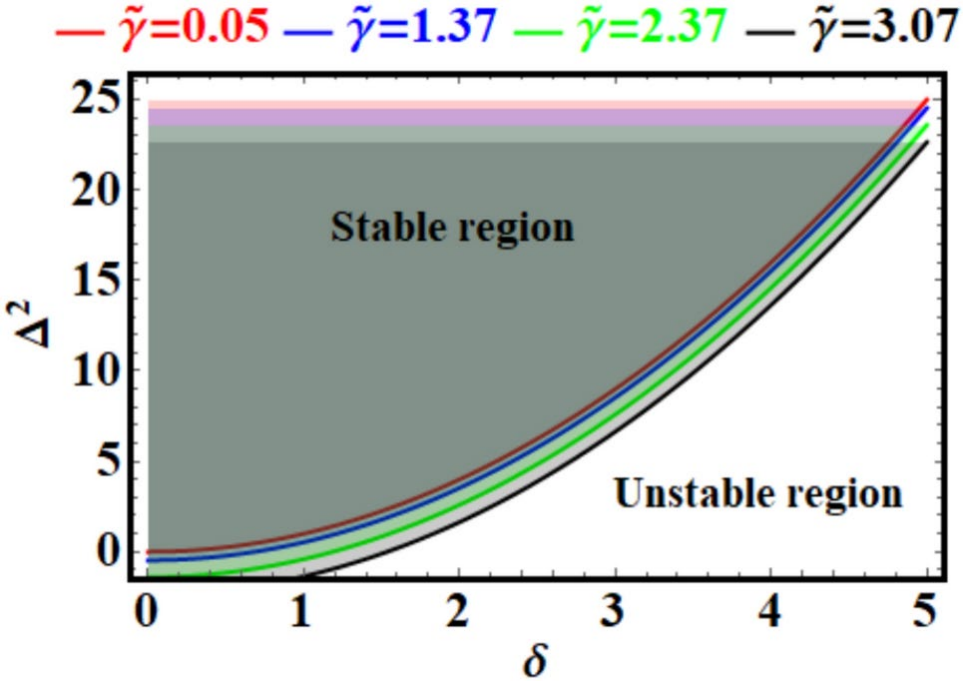
Reveals the stability curves for different values of $\widetilde{\gamma }$ in view of Eq. (24).

Figure 12 represents a stability map in the $\delta - \Delta^{2}$ parameter space. The system behavior is categorized into; a stable region that lies above the curves for different values of $\tilde{\gamma }$ (red, blue, green, and black lines) and the unstable one that is located below the curves, where the system dynamics become divergent or unbounded. The stability threshold $\Delta^{2}$ increases nonlinearly with $\delta$. Higher $\delta$ values require larger $\Delta^{2}$ to maintain stability. As $\tilde{\gamma }$ increases, the stability boundary shifts downward. This implies stronger damping ($\tilde{\gamma }$) reduces the required $\Delta^{2}$ for stability, effectively enlarging the stable region. At $\delta = 0$, all stability curves converge near the same $\Delta^{2}$ value, indicating the system is most stable at lower $\delta$. For higher $\delta$, stability depends significantly on $\tilde{\gamma }$, with larger $\tilde{\gamma }$ values offering greater robustness. Increasing $\delta$ pushes the system closer to instability, requiring higher $\Delta^{2}$ counteracting the destabilizing effects. Higher damping ($\tilde{\gamma }$) provides greater resilience to destabilizing factors, evidenced by the lower stability boundary. Stability and instability are essential ideas with significant implications across multiple disciplines, including physics, engineering, economics, and social systems. Stability denotes a system’s capacity to sustain or revert to equilibrium when faced with perturbations, hence assuring predictability, order, and resilience. This is crucial in structures, ecosystems, financial markets, and governance, as it facilitates sustained operation and progress. Instability denotes a system’s susceptibility to disturbances, occasionally resulting in swift alterations, disorder, or failure. Instability may present obstacles, although it can also catalyse innovation and transformation as systems adapt or evolve in reaction to external influences. These notions underscore the intricate equilibrium necessary to sustain harmony while promoting advancement in dynamic settings.

## Conclusion

An SP is a point mass that is suspended from a thread or rod and has a little amount of mass. It is a structure with a single resonance frequency. The usage of SPs occurs in a wide range of engineered objects, such as clocks, seismographic instruments metronomes, and entertainment at amusement parks, is generally acknowledged. The previous manuscript included three different problems, each of them were concerned with a SP. The first problem concerned with a charged magnetic spherical SP, the second pendulum was made of heavy cylinders that roll effortlessly in a plane that was horizontal while attached to a SP, and the latter one concerned a nonlinear dynamical model demonstrating the motion of a damped spring SP in a fluid flow. The NPA, as a novel methodology, was used to examine these problems. Simply said, the new strategy converted the nonlinear ODE into a linear one. The disappearance of Taylor expansion processing weakness was the main advantage of adopting the NPA. Therefore, the current restoration crowded has not received any upgrades. Second, the novel approach enabled us to examine the system’s stability requirements without forgoing any traditional perturbation methods. The novel approach was validated through numerical corresponding between the linear equation and the nonlinear equation utilizing the MS. The very low absolute inaccuracy that results was advantageous. The time history of the calculated solution and the associated phase plane plots were gathered in order to further demonstrate the influence of the influencing factors on the motion behavior. The two responses may be compared and a good fitting between them was seen, indicating the correctness of the approach. Several phase portraits were designed to highlight the various types of stability and instability near equilibrium points, where the relationship between the expanded and cyclotron frequencies (generated by the magnetic field) was examined for different values of azimuthal angular velocity. The subsequent outcomes should be emphasized on the original approach or significant results:

1. The given technique generated an additional ODE that was equivalent to a nonlinear one.
2. There was a strong correlation between these two equations.
3. In the presence of restoring forces, all conventional approaches utilized Taylor expansion to simplify the presented problem. This vulnerability was eliminated in the current strategy.
4. In contrast to previous conventional methods, the current methodology enabled us to examine the stability analysis of the problem.
5. In conclusion, it seemed that the present novel approach was a straightforward, functional, and attractive tool. It can be used to analyze many types of nonlinear oscillations.

It should be noted that there are two fundamental assumptions (constraints) in the management of Non-Performing Assets (NPA). The principal concerns relate to the starting conditions. The second one relates to the small magnitude of the starting amplitude to attain enhanced precision.

In subsequent paper, our aim is to analyze the SPs of multi-degrees of freedom in accordance of the following features:

1. The notion of multiple degrees of freedom in basic pendulums expands their traditional single-degree-of-freedom motion to intricate systems where several interconnected pendulums operate independently or interactively.
2. This greatly enhances their physical behavior, facilitating the modelling of complex dynamical systems seen in both natural and manmade environments.
3. Multi-degree-of-freedom pendulums are essential for examining linked oscillations, wave propagation, and energy transfer, with applications in mechanical and civil engineering, robotics, and seismology.
4. For instance, comprehending their dynamics might enhance vibration management in structures, refine the design of coupled oscillatory systems in machinery, or facilitate the advancement of sophisticated robotics with flexible joints.
5. Furthermore, they function as simplified representations of more intricate processes, including molecular vibrations in chemistry or chaotic systems in physics.
6. Through the examination of their degrees of freedom, researchers can acquire insights into resonance, stability, and energy distribution, rendering multi-degree-of-freedom pendulums essential for investigating nonlinear dynamics and creating novel solutions.
7. It is preferable to extend the NPA to encompass solitons and their stability analysis^45–48^.

Across all the phase plane diagrams in Figs. 3, 7, and 11, the general implications can be interpreted as providing a valuable framework for understanding stability, chaos, and periodicity, phenomena common to many systems in nature and engineering. Furthermore, oscillatory and spiral behaviors captured in these diagrams provide insights into energy dynamics such as conservation, dissipation, and transfer. These tools are essential for engineers aiming to optimize system performance for stability and efficiency.

## Data Availability

All data generated or analyzed during this study are included in this published article.

## References

[1] Moatimid, G. M. & Amer, T. S. Analytical approximate solutions of a magnetic spherical pendulum: Stability analysis. *J. Vib. Eng. Technol.* **11**, 2155–2165 (2023).

[2] Moatimid, G. M. & Amer, T. S. Analytical solution for the motion of a pendulum with rolling wheel: Stability analysis. *Sci. Rep.* **12**, 12628 (2022). 35871675 10.1038/s41598-022-15121-w PMC9309161

[3] Moatimid, G. M., Amer, T. S. & Zekry, M. H. Analytical and numerical study of a vibrating magnetic inverted pendulum. *Arch. Appl. Mech.* **93**, 2533–2547 (2023).

[4] Montwiłł, A., Kasińska, J. & Pietrazak, K. Importance of key phases of the ship manufacturing system for efficient vessel life cycle management. *Procedia Manuf.* **19**, 34–41 (2018).

[5] Moatimid, G. M., El-Sayed, A. T. & Salman, H. Dynamical analysis of an inverted pendulum with positive position feedback controller approximate uniform solution. *Sci. Rep.* **13**(1), 8849 (2023). 37258590 10.1038/s41598-023-34918-x PMC10232474

[6] Elbori, A. & Abdalsmd, L. Simulation of simple pendulum. *Int. J. Eng. Sci. Invent.* **6**(4), 33–38 (2017).

[7] Mohazzabi, P. & Shankar, S. Damping of a simple pendulum due to drag on its string. *J. Appl. Math. Phys.* **5**, 122–130 (2017).

[8] Azizahwati, A., Rahmad, M. & Zamri, R. Development of digital simple pendulum learning media. *J. Phys: Conf. Ser.* **2049**, 012065 (2021).

[9] Chen, Z., Wang, Y., Wu, Q. & Cui, J. Investigating the drag impact on pendulum through numerical models and the stability analysis. *Theor. Nat. Sci.* **43**(1), 286–297 (2024).

[10] Bhakat, P., Chakraborty, S. & Mandal, P. Tracking the motion of a simple pendulum with tracker. *Resonance* **29**, 1085–1093 (2024).

[11] Guckenheimer, J. & Holmes, P. *Nonlinear oscillations, Dynamical Systems, and Bifurcations of Vector Fields, Applied Mathematical Sciences (AMS volume 42)* (Springer, 1983).

[12] Kontomaris, S. V., Mazi, I. & Malamou, A. A note on a simple equation for solving nonlinear undamped oscillations. *J. Vib. Eng. Technol.* **12**, 8235–8248 (2024).

[13] Watamura, T., Iwata, R. & Sugiyama, K. Free surface oscillation driven by rotating stirrer. *Eur. Phys. J. E* **47**, 26 (2024). 38613716 10.1140/epje/s10189-024-00420-z PMC11249497

[14] Hasil, P., Linhartová, L. & Veselý, M. Oscillation criterion for generalized Euler difference equations. *Acta Math. Hungar.* **174**, 94–115 (2024).

[15] Fahimi, K., Mädler, L. & Ellendt, N. Exploring droplet oscillation dynamics in surface tension measurements. *Exp. Fluids* **65**, 184 (2024).

[16] Ponomarev, A., Hagenmeyer, V. & Gröll, L. Nonlinear analysis of the synchronous reference frame phase-locked loop under unbalanced grid voltage. *Nonlinear Dyn.* **112**, 9225–9243 (2024).

[17] Sergeev, I. N. Study of the complete oscillation, rotation, and wandering properties of a differential system by the first approximation. *Math. Notes* **115**, 599–606 (2024).

[18] Petrov, A. G. Nonlinear forced oscillations of a bubble in a liquid. *Dokl. Phys.* **68**, 267–271 (2023).

[19] Amer, A., Zhang, W. & Amer, T. S. Parametric excitation and chaos in nonlinear forced Mathieu system: A comprehensive analysis. *Alex. Eng. J.* **116**, 35–54 (2025).

[20] He, J.-H. Comment on He’s frequency formulation for nonlinear oscillators. *Eur. J. Phys.* **29**, L19–L22 (2008).

[21] He, J.-H. An improved amplitude-frequency formulation for nonlinear oscillators. *Int. J. Nonlinear Sci. Numer. Simul.* **9**(2), 211–212 (2008).

[22] Geng, L. & Cai, X.-C. He’s frequency formulation for nonlinear oscillators. *Eur. J. Phys.* **28**(5), 923–931 (2007).

[23] Ren, Z.-F. & Wu, J.-B. He’s frequency–amplitude formulation for nonlinear oscillator with damping. *J. Low Freq. Noise, Vib. of Active Control* **38**(3–4), 1045–1049 (2019).

[24] Ghaleb, A. F., Abou-Dina, M. S., Moatimid, G. M. & Zekry, M. H. Analytic approximate solutions of the cubic-quintic Duffing Van der Pol equation with two-external periodic forces terms: Stability analysis. *Math. Comput. Simul.* **180**, 129–151 (2021).

[25] Moatimid, G. M. Sliding bead on a smooth vertical rotated parabola: Stability configuration. *Kuwait J. Sci.* **47**(2), 6–21 (2020).

[26] Moatimid, G. M., El-Dib, Y. O. & Zekry, M. H. Stability analysis using multiple scales homotopy approach of coupled cylindrical interfaces under the influence of periodic electrostatic fields. *Chin. J. Phys.* **56**(5), 2507–2522 (2018).

[27] Moatimid, G. M. & Amer, T. S. Analytical solution for the motion of a pendulum with rolling wheel: Stability Analysis. *Sci. Rep.* **12**(1), 12628 (2022). 35871675 10.1038/s41598-022-15121-w PMC9309161

[28] Ren, Z. & Hu, G. He’s frequency–amplitude formulation with average residuals for nonlinear oscillators. *J.Low Freq. Noise, Vib. Active Control* **38**(3–4), 1050–1059 (2019).

[29] Ren, Z. A simplified He’s frequency–amplitude formulation for nonlinear oscillators. *J. Low Freq. Noise, Vib. Active Control* **41**(1), 209–215 (2022).

[30] He, C. & Liu, C. A modified frequency-amplitude formulation for fractal vibration system. *Fractals* **30**(03), 2250046 (2022).

[31] Ismail, G. M., Moatimid, G. M. & Yamani, M. I. Periodic solutions of strongly nonlinear oscillators using He’s frequency formulation. *Eur. J. Pure Appl. Math.* **17**(3), 2154–2171 (2024).

[32] Moatimid, G. M. & Amer, T. S. Dynamical system of a time-delayed -Van der Pole oscillator: A non-perturbative approach. *Sci. Rep.* **13**, 11942 (2023). 37488150 10.1038/s41598-023-38679-5 PMC10366103

[33] Moatimid, G. M., Amer, T. S. & Ellabban, Y. Y. A novel methodology for a time-delayed controller to prevent nonlinear system oscillations. *J. Low Freq. Noise, Vib. Active Control* **43**(1), 525–542 (2024).

[34] Moatimid, G. M., Amer, T. S. & Galal, A. A. Studying highly nonlinear oscillators using the non-perturbative methodology. *Sci. Rep.* **13**, 20288 (2023). 37985730 10.1038/s41598-023-47519-5 PMC10662384

[35] Moatimid, G. M., El-Sayed, A. T. & Salman, H. F. Different controllers for suppressing oscillations of a hybrid oscillator via non-perturbative analysis. *Sci. Rep.* **14**, 307 (2024). 38172592 10.1038/s41598-023-50750-9 PMC10764832

[36] Alluhydan, K., Moatimid, G. M. & Amer, T. S. The non-perturbative approach in examining the motion of a simple pendulum associated with a rolling wheel with a time-delay. *Eur. J. Pure Appl. Math.* **17**(4), 3185–3208 (2024).

[37] Alluhydan, K. et al. A novel inspection of a time-delayed rolling of a rigid rod. *Eur. J. Pure Appl. Math.* **17**(4), 2878–2895 (2024).

[38] Moatimid, G. M., Mohamed, M. A. A. & Elagamy, Kh. An innovative approach in inspecting a damped Mathieu cubic–quintic Duffing oscillator. *J. Vib. Eng. Technol.* **12**(Suppl 2), S1831–S1848 (2024).

[39] Moatimid, G. M. & Amer, T. S. Inspection of some extremely nonlinear oscillators using an inventive. *J. Vib. Eng. Technol.* **12**(Suppl 2), S1211–S1221 (2024).

[40] Moatimid, G. M., Mohamed, M. A. A. & Elagamy, Kh. Insightful examination of some nonlinear classification linked with Mathieu oscillators. *J. Vib. Eng. Technol.* **13**, 173 (2025).

[41] Alanazy, A., Moatimid, G. M., Amer, T. S., Mohamed, M. A. A. & Abohamer, M. K. A novel procedure in scrutinizing a cantilever beam with tip mass: Analytic and bifurcation. *Axioms* **14**, 16 (2025).

[42] Ismail, G. M., Moatimid, G. M., Alraddadi, I. & Kontomaris, S. V. Scrutinizing highly nonlinear oscillators using He’s frequency formula. *Sound Vibration* **59**(2), 2358 (2025).

[43] Alanazy, A., Moatimid, G. M. & Mohamed, M. A. A. Innovative methodology in scrutinizing nonlinear rolling ship in longitudinal waves. *Ocean Eng.*10.1016/j.oceaneng.2025.120924 (2025).

[44] Bek, M. A., Amer, T. S., Sirwah, M. A., Awrejcewicz, J. & Arab, A. A. The vibrational motion of a spring pendulum in fluid flow. *Res. Phys.* **19**, 103465 (2020).

[45] Ali, A., Ahmad, J., Javed, S. & Rehman, S.-U. Exact soliton solutions and stability analysis to (3 + 1)-dimensional nonlinear Schrödinger model. *Alex. Eng. J.* **76**, 747–756 (2023).

[46] Ali, A., Ahmad, J. & Javed, S. Exploring the dynamic nature of soliton solutions to the fractional coupled nonlinear Schrödinger model with their sensitivity analysis. *Opt. Quant. Electron.* **55**, 810 (2023).

[47] Ali, A., Ahmad, J., Javed, S., Alkarni, S. & Shah, N. A. Investigate the dynamic nature of soliton solutions and bifurcation analysis to a new generalized two-dimensional nonlinear wave equation with its stability. *Res. Phys.* **53**, 106922 (2023).

[48] Javed, S., Ali, A., Ahmad, J. & Hussain, R. Study the dynamic behavior of bifurcation, chaos, time series analysis and soliton solutions to a Hirota model. *Opt. Quant. Electron.* **55**, 1114 (2023).

